# Perceptions of nurse educators and nursing students on the model for facilitating ‘presence’ in large class settings through reflective practices: a contextual inquiry

**DOI:** 10.1186/s12912-023-01341-6

**Published:** 2023-05-26

**Authors:** Kathleen Froneman, Emmerentia du Plessis, Anna Catharina van Graan

**Affiliations:** Faculty of Health Sciences, School of Nursing Science, North-West University, Private Bag X1290, Potchefstroom, 2520, Cell 0834822503 South Africa

**Keywords:** Large class settings, Nurse educators, Nursing students, Presence, Reflective practices

## Abstract

**Background:**

Nursing education starts in the classroom environment with a focus on the nurse educator-nursing student relationship. ‘Presence’ is defined as “a practice where the caregiver relates her/himself to the other in an attentive and dedicated way, by doing so learns to see what is at stake for the other; from desires to fear, and, in connection with this, come to understand what could be done in this particular situation and who she/he can be for the other”. ‘Presence’ forms an integral part of the nursing profession and the value thereof should be facilitated during teaching and learning. Reflective practices may offer a teaching–learning strategy to facilitate presence in nursing students by nurse educators in large class settings. Having large classes presents challenges including from nurse educators’ lack of knowledge about alternative teaching approaches; time demands for designing, implementing and testing new teaching methods; a lack of confidence in implementing new teaching approaches in the classroom; selecting and grading assessments; as well as feelings of discomfort and anxiety. A model to facilitate presence through reflective practices has already been developed and published by the present authors. The model relies on well-established steps in theory development covering concept analysis, model development and description (published in two papers by the present researchers) and model evaluation (the subject of this paper). The evaluation was carried out by a panel of experts and nursing participants.

**Methods:**

An explorative and descriptive qualitative design was followed. The developed model was evaluated and refined in two steps (covered in this paper). In Step 1, the model was evaluated by a panel of experts in model development, reflective practices and presence. The panel used critical reflection resulting in the refinement of the model. Step 2 involved an empirical phase where the model was evaluated by participants through participatory evaluation. Participants were selected through purposive sampling. Data collection methods included online semi-structured focus group interviews with nurse educators and virtual World Café sessions with nursing students. Content analysis was done through open coding.

**Results:**

Five themes emerged from the empirical phase, namely: Theme 1: understanding of the model; Theme 2: benefits of the model; Theme 3: limitations of the model; Theme 4: pre-existing conditions needed for successful implementation of the model; and Theme 5: recommendations for further development of the model.

**Conclusions:**

The results produced a refined model to be implemented into the curriculums of undergraduate, postgraduate and continuous professional development programmes across nursing education institutions. This model will significantly contribute to the body of knowledge and increase nurses’ awareness of presence by transforming the way they feel, think, care and act in practice, which contributes to personal and professional development.

## Background

In South Africa, nursing education is specifically directed at the development of the nursing student as an adult learner. This development should take place on a personal and professional level and should lead to the cognitive, affective and psychomotor development of the nursing student, as well as the achievement of the prescribed programme outcomes [1]. It has furthermore been stated by the South African Nursing Council (SANC) [2] that in nursing education, the responsibility of the nurse educator is the development, content, coordination, presentation and control of the specific nursing education programme.

Quality nursing care embraces ‘presence’ as one of the essential components that lead to increased patient satisfaction [3, 4]. ‘Presence’ is defined as “a practice where the caregiver relates her/himself to the other in an attentive and dedicated way, by doing so learns to see what is at stake for the other; from desires to fear, and, in connection with this, come to understand what could be done in this particular situation and who she/he can be for the other” [5]. As stated by Du Plessis and Beurskens, “presence has a close link with quality nursing care and patient satisfaction, as it is about understanding. When practising presence, reflecting on the patient’s understanding of the situation brings perspective and leads to mutual understanding and a moment of connection and appropriate action” [6]. Through cultivating critical thinking and the use of reflective practices, presence can be developed [7]. Furthermore, critical thinking and reflective practices are specific skills associated with nursing as a profession [2]. Therefore, nurses are required to be critical thinkers and need to be encouraged by nurse educators to practice presence while questioning and reflecting daily on their practice.

Presence in the context of the nurse educator–nursing student relationship is about understanding, as well as being open to the reasoning and frame of reference of both the nurse educator and nursing student. Presence does not often feature explicitly in nursing education programmes. International and national literature accentuate reflective practices as among the most suitable teaching–learning strategies to facilitate presence in nursing students [7, 8]. This approach can be significant in large class settings where nursing students can easily become ‘a number’ and where it is difficult to engage with them individually. Moreover, teaching–learning through reflective practices may be a challenge for nurse educators because it is a difficult and time-consuming skill.

The COVID-19 pandemic further contributes to this challenge where teaching–learning was adapted from face-to-face teaching to virtual teaching, resulting in the modification of teaching–learning practices. Therefore, it can be viewed by nurse educators as an additional expectation to their already challenging workload of managing large class groups and balancing the demands of teaching, clinical supervision and research, while still sustaining quality interaction with nursing students [9, 10]. In addition, nurse shortages in South Africa, lead to an increased number of nursing students being trained. Training of large class groups may affect the standard and quality of nursing education as well as the teaching–learning strategies nurse educators use and therefore the experience of nursing students. A model for nurse educators to facilitate their presence in large class groups of nursing students through reflective practices, was developed and published by the present authors [11].

This article aimed to evaluate and refine a developed model for nurse educators to facilitate their presence in large class settings through reflective practices using theoretical evaluation by a panel of experts; and participatory evaluation by nursing participants.

## Methods

### Study design

A qualitative, explorative, descriptive and contextual design was followed. The model was evaluated and refined in two steps. In Step 1, the model was evaluated by a panel of experts using critical reflection resulting in the refinement of the model. Step 2 involved an empirical phase where the model was evaluated by participants through participatory evaluation to explore and describe the perceptions of nurse educators and nursing students [10]. This design was deemed appropriate as there was a need to know how nurse educators and nursing students perceived the model for nurse educators to facilitate presence in large class settings through reflective practices as a teaching–learning strategy.

### Study setting

The North West Province is one of the provinces with the highest number of nursing students in training in relation to the total number of accredited nursing education institutions (NEIs) that provide the 4-year undergraduate nursing programme. There are 10 SANC-accredited NEIs within the North West Province. Six of these are public NEIs and four are private NEIs. Only two public accredited NEIs were included in this study as none of the other NEIs offer the 4-year undergraduate nursing programme.

### Evaluation by a panel of experts

#### Sample

Typical case purposive sampling was used to select experts in the field of nursing with specialist knowledge relevant to the study [12]. The inclusion criteria for the panel of experts included holding a doctoral degree; being recognised as knowledgeable in higher education, nursing education, presence and reflective practices; and/or experience of model development by others or through scientific publications; and having nationally and internationally recognised profiles in the different fields as listed above. Seven people were invited to become panel members, four of whom participated in the theoretical evaluation.

#### Data collection

Each panel member received an electronic copy of the description and graphic presentation of the model and an evaluation form developed from the literature [13]. A panel discussion was scheduled via Zoom. The model was presented to the panel of experts through a PowerPoint presentation and was followed by a discussion session. Panel members (identified by numbers) were allowed to ask questions and clarify any uncertainties, whereafter they completed and submitted the evaluation form. The evaluation form contained two sections. Section A of the evaluation form included the demographic data of each panel member and Section B contained the criteria for critical reflection.

#### Data analysis

Critical reflection contributes to understanding of how well the model relates to practice, research or educational activities [13]. The five generic questions, criteria and description used to evaluate the model were included in the evaluation form as summarised in Table 1.

**Table 1:** Critical reflection for evaluation of the model (Chinn & Kramer, 2018)

QUESTION	DESCRIPTION
How clear is this model?	For clarity, the model must comply with four criteria, namely: semantic clarity, semantic consistency, structural clarity and structural consistency. Thus this question addresses the clarity and consistency of the model from both semantic and structural perspectives.
How simple is the model?	This question addresses the structural components and relationships within the model. It can include complexity referring to numerous components in the model, and simplicity implying fewer relational components.
How general is this model?	This question addresses the purpose and the scope of experiences covered by the model. Generality refers to a wide scope of phenomena whereas specificity narrows the range of events.
How accessible is the model?	This question addresses the extent to which concepts within the model are grounded in empirically identifiable phenomena.
How important is the model?	This question addresses the extent to which the model leads to valued nursing goals in practice, research and education.

The criteria provided to participants for the evaluation of the model included participants’ perceptions of the model’s clarity, simplicity, generality, accessibility, and importance. Feedback from the panel is summarised in Table 2.

**Table 2:** Section B: Evaluation of the model

CRITERIA	NOT ACCEPTABLE OR NEEDS MAJOR CHANGES	ACCEPTABLE WITH RECOMMENDED CHANGES	ACCEPTABLE AS DESCRIBED	COMMENTS
**Model Validation N = 4**				
**1. Clarity of the model**				
a) *Semantic clarity*: • Are the concepts clearly defined? • Are the definitions understandable and coherent?	0 (0%)	1 (25%)	3 (75%)	• Clear definitions from literature and subject definitions are provided and are understandable. • reconsider the word ‘transformational’ for better characterisation of the learning process.
b) *Semantic consistency*: • Are the concepts congruent and in harmony with the definitions and purpose and aligned to the relationships featured in the model?	0 (0%)	2 (50%)	2 (50%)	• Provide a network view of the involved concepts (not the too-detailed lists). • Transformational learning in the current format is presented as a linear and one-dimensional manner and therefore leads to missing the dynamic nature thereof and the relationship.
c) *Structural clarity*: • Are the illustrated connections and logical reasoning coherent with the descriptive elements of the model?	1 (25%)	3 (75%)	0 (0%)	• To characterise the lines and arrows more specifically: is cause of…, is impetus to …, is effect of…, is co-occurring with…, is part of…, is condition to … etc”; and “change the process structure because it is less a closed circle and more an endless spiral”. • Change of structure, landscape and dimensional. • I missed this in the description of the model. The one-directional nature of transformation learning seems at present as an action activated by the lecturer to influence the learner, whilst it can rather be considered that the intense and complex relationship between the lecturer and student in which transformational learning is facilitated, rather presents an interactive and reciprocal effect. • Look at the flow of how the model is arranged.
d) *Structural consistency*: • Do the structural forms used for illustration as a conceptual map enhance the clarity and comprehension of the descriptive elements of the model?	1 (25%)	2 (50%)	1 (25%)	• A sketch of the starting position of the students. Students are not recipients but co-creators with their own input and developments. • The clouds in the middle could be replaced by the dynamics as these exist between the student and nurse educator. • Illustrate the interactivity between the agent and recipient and the process and outcomes of this transformational learning.
**2. Simplicity of the model**:				
a) Are the number and differentiation of concepts and interrelationships least in simplicity or acceptable in complexity?	1 (25%)	1 (25%)	2 (50%)	• Love the simplicity. • The background consists of complex practices, mandatory rules and regulations, instable politics (also the insurances companies), legal requirements, etc. It is not such a simple and peaceful background as described in the text”; and “The text is overloaded with details and extended enumerations: being more selective, less redundant and more frequently zooming out make your model stronger. Less is more! • Present the depth, multidimensional nature of the concepts with the context of large classes, fitting a practice model, here in South Africa. • Use less text for the model. Simple is key and only relevant concepts are represented in the model. • The preferred learning styles – see literature. • The essential competences of the teacher are listed: the competence ‘able to teach’ is missing. • The phasing of the educational process: how do people learn, in which sequences? • Better characterisation of the learning process is needed.
b) Does the contextual situation warrant the various concepts to enhance understanding of the concepts and their interrelatedness in the model?	0 (0%)	2 (50%)	2 (50%)	
c) Does the model serve to describe, explain and/or predict concepts or their interrelatedness in practice?	0 (0%)	2 (50%)	2 (50%)	
**3. Generality of the model**:				
a) Do the breadth of scope and specificity of purpose appraise the broad empirical experiences of concepts for the purpose of nursing?	0 (0%)	2 (50%)	2 (50%)	• Love this- value and simplicity. • A list of intended competencies (or: outcome – the blue banner is not enough in this respect). • Being a practice model, reading through the description, the theoretical justification for the model was present but the tangible practical application of presence seemed absent – how to do reflective practices for presence specially in the large-class setting. • Arrange the process and flow of the model so that is easy to understand. For example, the dynamics appear as if they are a result of what is currently happening in the model. A suggestion is that an outcome is highlighted. The outcome is nursing student who is present and is reflective. • We need to address social justice.
b) Are ideas arranged to facilitate application to practice and the health care team while embodying nursing as a discipline?	0 (0%0	2 (50%)	2 (50%)	
c) Are the concepts of the individual, health, environment and society featured broadly in the general application of the model?	0 (0%)	3 (75%)	1 (25%)	
**4. Accessibility of the model**:				
a) Would the concepts be identified as empirical indicators in practice within the realm of nursing?	1 (25%)	0 (0%)	3 (75%)	• The large class realities in nursing education which includes in the South African context also diversity brings the contextual realities in which the reflective practices for presence are to be facilitated. In addition, large classes bring forth various challenges that impact especially this relationship between the lecturer and student. I missed the practical application of the model onto these realities.
b) Do the definitions of the concepts adequately manifest their meanings in the nursing practice setting that is specified?	0 (0%)	1 (25%)	3 (75%)	
c) Despite either the simplicity or complexity of the model, do the concepts create conceptual meanings in the clinical practice setting?	0 (0%)	1 (25%)	3 (75%)	
**5. Importance of the model**:				
a) Does the model have clinical value or practical significance in the targeted area of clinical nursing practice?	0 (0%)	0 (0%)	4 (100%)	• The too high and too encompassing standards undermine the model: they cannot and will not be met, and the conclusion will be that presence cannot be taught nor learned, at least not in this way, if ever. That would be harmful
b) Is there futuristic and pragmatic value in the applicability to lead future practice of nursing in the targeted area?	0 (0%)	1 (25%)	3 (75%)	
c) Does the theory in the model create understanding and the potential for nursing education and research?	0 (0%)	0 (0%)	4 (100%)	
d) Does the model differentiate the focus or nature of nursing as a discipline separate to other service professions?	0 (0%)	0 (0%)	4 (100%)	
**General**:				
a) Importance for research, practice and education	0 (0%)	1 (25%)	3 (75%)	• I can gladly confirm that you have reached the point of presenting a practice model • The final challenge now is for you to present the depth, multidimensional nature of the concepts with the context of large classes, fitting a practice model, here in South Africa
b) Validity or trustworthiness	1 (25%)	0 (0%)	3 (75%)	
c) Other (Specify)	0 (0%)	1 (25%)	3 (75%)	

### Evaluation by participants

#### Sample

The population for the empirical phase comprised n = 38 nurse educators and n = 34 nursing students from N = 4 research sites. These sites included a university and nursing college with two campuses each all of which are accredited nursing education institutions (NEIs) offering the 4-year undergraduate nursing programme within the North West Province. Typical case purposive sampling was used because the participants were selected to explore and describe how nurse educators and nursing students at accredited NEIs within the North West Province perceive, interpret and understand the model. Inclusion and exclusion criteria with their rationale for nurse educators as participants for online semi-structured focus group interviews and nursing students as participants for virtual World Café sessions are summarised in Table 3.

**Table 3:** Inclusion and exclusion criteria for nurse educators and nursing students

**Nurse educators as participants in online semi-structured focus group interviews**	
**Inclusion criteria**	**Rationale**
• Participants must have at least 2 years of experience as nurse educators in accredited NEIs.	• Both presence and reflective practices require expertise and in-depth practice; it is an art that needs to be developed over time.
• Participants must be involved in teaching and learning of nursing students enrolled in the 4-year undergraduate nursing programme.	• The nurse educator is responsible for teaching undergraduate nursing students who are the future professional nurses entering their nursing career as competent, reflective practitioners being present during nursing care.
• Participants must be proficient in English.	• Data collection was conducted in English.
**Exclusion criteria**	**Rationale**
• Participants who are only involved in teaching and learning of the postgraduate nursing programme.	• Postgraduate programme involves students who are registered nurses and already supposed to practice presence.
• Participants involved in research and who have no teaching responsibilities.	• These participants function in research positions and are not involved in teaching and learning.
• Participants involved in management, e.g., the Director of the Nursing School at the University and the Principal of the Nursing College.	• These participants are in managerial positions and not involved in teaching and learning.
**Nursing students as participants in virtual World café sessions**	
**Inclusion criteria**	**Rationale**
• Participants must be nursing students in their 4th year of training in the 4-year undergraduate nursing programme by the time data collection takes place.	• These participants will have gained the relevant and needed experience of being taught by a nurse educator during the previous 3 years and could provide more in-depth information.
• Participants must be proficient in English.	• Data collection was conducted in English.
**Exclusion criteria**	**Rationale**
• Participants who are enrolled in the 4-year undergraduate nursing programme and in their 1st, 2nd and 3rd year of training by the time data is collected.	• These participants will possibly not have the necessary experience to inform the purpose of the study.

#### Data collection

Data was collected in two stages. In Stage 1, online semi-structured focus group interviews were conducted with nurse educators involved with the teaching and learning of undergraduate nursing students enrolled in the 4-year undergraduate nursing programme at accredited NEIs. During Stage 2, virtual World Café sessions were held with nursing students in their 4th year of nursing training.

##### Informed consent

Informed consent was obtained from all the participating nurse educators and nursing students.

##### Stage 1: online semi-structured focus group interviews.

Online semi-structured focus group interviews with nurse educators. were set up to facilitate the sharing of experiences, perceptions, ideas, feelings and viewpoints among the group participants [14, 15]. The use of online semi-structured focus group interviews was beneficial to participants, providing them with a convenient and comfortable way of participating in the discussion [16]. Six online semi-structured focus group interviews were conducted with n = 38 nurse educators lasting 90–110 min. Nurse educators were between the ages of 29 and 66 years. The majority of participants were female. The majority of participants have an additional qualification in nursing education with 2–22 years of teaching–learning experience. Groups included a maximum of six to eight participants to ensure that all participants had the opportunity to participate [15]; the small group size also enhanced the discussion and interaction (especially when participants had shared similar experiences held analogous views, and felt that they had a lot in common [15, 17]. The study design enabled data saturation to be attained [16].

The focus group interviews were initiated by welcoming the participants and providing them with a brief introduction to the purpose of the research, setting some basic ground rules, emphasising active participation, and reassuring participants regarding shared confidentiality and anonymity of the information [14]. The model was presented to participants via a PowerPoint presentation. An interview protocol outlined in Table 4 was applied [15].

**Table 4:** Interview schedule

**Interview questions used during online semi-structured focus group for nurse educators**
**Engagement/opening question**: Please introduce yourself and tell me about any special memory of being a nurse educator. **Introductory question**: How would you explain the model in your own words? **Exploration/key questions**: 1 Will you implement this model in your classroom and how will this influence your teaching practices? 2 How do you think this model will benefit you and your nursing students if implemented? 3 Please explain situations where you would apply this model. 4 What conditions need to exist to implement this model? 5 In your view, what are the limitations of this model? 6 From your experience, what can be included to make this model more useful, relevant and effective? **Exit/ending question**: Is there anything else or any further comments regarding this topic that you would like to add?
**Interview questions used during virtual World Café for nursing students**
**Exploration/key questions**: 1 What did you understand from the model presented to you? 2 What are the main ideas of the model? 3 In your opinion, what will the advantages be if this model is implemented in the classroom? 4 In your opinion, what will the disadvantages be if this model is implemented in the classroom? 5 From your experience, what can be included to make this model more useful, relevant and effective?

##### Stage 2: virtual World Café sessions.

The World Café is a living network of conversations for collaborative dialogue, sharing knowledge and creating possibilities for action in groups of nursing students around specific questions [19, 20]. It is seen as a brainstorming tool to generate ideas and comments about nursing students’ perceptions of the model to facilitate presence in large class settings through reflective practices as a teaching–learning strategy. Three virtual World Café sessions with n = 34 nursing students were conducted lasting 60–90 min. Nursing students are regarded as vulnerable thus participants needed to already be 4th-year students, all of whom are enrolled at NEIs within the North West Province. Data saturation occurred with the 3rd World Café, i.e., no new ideas, information and themes within a specific group were added or repeated [18].

During the introduction, all participants were welcomed and thanked for their participation. The model was presented via a PowerPoint presentation. Participants moved into breakout rooms assigned to them. In the breakout room, participants had five minutes to discuss the first question amongst themselves. After five minutes, they returned to the main meeting room. At the end of each conversational round, the individual groups returned to the main meeting where a hyperlink was provided with the question in the chat room. When participants clicked on the hyperlink, they were guided to the Mentimeter app, displaying the question to be addressed. Participants were provided with space to write down numerous entries from their respective points of view whereafter they needed to click ‘submit’. Each round of questions happened in the same way and continued until all questions were answered. The complete list of discussion questions is outlined in Table 4.

In the discussion session, the host shared each ‘tablecloth’ in the main meeting room for all participants to see the results that had been generated and to provide participants with an opportunity to elaborate on their answers or clarify any misunderstanding.

#### Field notes

Field notes were taken for clarification purposes and were reflected on during data analysis. Field notes were compiled directly after each data collection method had been applied and included methodological notes (incorporating reflections on the method and strategies), theoretical notes (based on own thoughts and reflections) and personal notes (based on own feelings to verify and enrich the findings) [14, 18].

#### Data analysis

Data analysis confirmed participants’ understanding [17] of the model. The process of open co-coding through content analysis was followed to allow research findings to emerge from frequent, dominant or significant themes inherent in the raw data without the constraints imposed by a more structured theoretical orientation [21]. Creswell’s content analysis involved a linear, hierarchical, interactive approach, building from the bottom to the top, which was appropriate to the purpose of this research study. An independent co-coder who is a known expert in the field of qualitative data analysis assisted with data analysis. Data was organised and prepared for analysis by transcribing each focus group interview and World Café session separately and was coded based on the following steps.

- Transcripts were read carefully. Ideas that came to mind were written down in the margin of the transcript.
- The most interesting and shortest transcript near the top of the pile was picked. It was read through while considering the question ‘what is it about?; The underlying meaning was reflected on, and thoughts were written down in the margin of the transcript.
- The remaining transcripts were read through using the same method.
- A list of all the topics that came to mind was developed. These topics were placed into columns, e.g., major topics, unique topics and leftovers.
- The list was taken back to the data. Topics were abbreviated using codes next to the appropriate segments of the text to see if new codes emerged.
- The most descriptive words for topics were found and turned into categories. Categories that related to each other were grouped together, and lines were drawn between categories to show interrelationships.
- A final decision on the abbreviation for each category was made and the categories were then placed in alphabetical order.

Themes and sub-themes were then generated. The field notes were reflected on and compared with the findings. A meeting was scheduled and attended by the coder and co-coder to reach consensus on the themes and sub-themes that emerged from the data collected. The themes and sub-themes for Stages 1 and 2 were further synthesised by the coder and co-coder by clustering similar and repeated themes and sub-themes together to form a combined whole.

#### Informed consent

Written informed consent was signed by participants who participated in this study. Participation was voluntary and participants gave consent to the use of data through their signed participation.

## Results

Five themes emerged and include (1) understanding of the model, (2) benefits of implementing the model, (3) limitations of the model, (4) pre-existing conditions needed for successful implementation of the model, and (5) recommendations for further development of the model. Table 5 provides an overview of the findings.

**Table 5:** Themes and sub-themes for evaluation of the model by nurse educators and nursing students

**THEME 1: UNDERSTANDING OF THE MODEL**			
**Sub-theme 1.1: Feasibility for implementation of the practice model**			
• Attributes of the nurse educator • Willingness and openness of nurse educators • Adapt new teaching methods • Adequate training • Sufficient time • Invest in proper planning			
**THEME 2: BENEFITS OF IMPLEMENTING THE MODEL**			
**Sub-theme 2.1: Benefits for nurse educators**	**Sub-theme 2.2: Benefits for nursing students**		**Sub-theme 2.3: Benefits for all stakeholders**
• Reflection helps to understand nursing students and if they are coping • Better collaboration • Enhance the quality of teaching (feedback from nursing students on nurse educators’ teaching practices) • Leading and empowering students • Creating interesting classrooms	• Students learn about their learning • Active participants in learning • Enhances involvement and interaction • Enables students to become lifelong learners • Ensures quality nurses • Becoming a critical thinker • Becoming an independent, creative and innovative practitioner • Ability to implement evidence-based practices • Improve decision-making skills • Integrating theory-practice		• Empowerment of nurse educators and nursing students • Improve relationships and collaboration • Improve the mental well-being of nurse educators and nursing students • Improve the quality of care • Bring presence to the forefront
**THEME 3: LIMITATIONS OF THE MODEL**			
**Sub-theme 3.1: Limited time**	**Sub-theme 3.2: Lack of resources**		**Sub-theme 3.3: Resistance to change**
• Time-consuming • Class time frames	• Insufficient equipment • Shortage of staff • Lack of support from NEIs • No devices • Large classes		• Struggle to change or to adapt to new practices
**THEME 4: PRE-EXISTING CONDITIONS NEEDED FOR SUCCESSFUL IMPLEMENTATION OF THE MODEL**			
**Sub-theme 4.1: Nursing education environment**	**Sub-theme 4.2: Staff development programmes**		**Sub-theme 4.3: Stakeholder collaboration and support**
• Classroom • Clinical practice during clinical accompaniment • Community	• In-service training of nurse educators on the model • Continuous follow-up with nurse educators after in-service training to encourage continuation		• Nurse educators to be invested • Positive attitudes from lecturers • Stakeholders’ buy-in
**THEME 5: RECOMMENDATIONS FOR FURTHER DEVELOPMENT OF THE MODEL**			
**Sub-theme 5.1: Theory-practice integration**		**Sub-theme 5.2: Inclusion of other stakeholders**	
• Implement the model in the practical setting		• Reflection of patient/s • Involvement of parent/s • Community of nurses	

### Theme 1: understanding of the model

The model was presented to participants whereafter they were asked to ‘explain the model in their own words’ to determine their understanding of the model. Participants demonstrated a good understanding of the main ideas and its concepts captured in the model and which enable the model to be implemented. Feasibility of implementing the model emerged as a sub-theme.

During the introductory session, nurse educators were asked to share ‘any special memory of being a nurse educator’. Participants’ responses reflected specific attributes of a nurse educator, which included being appreciated, adding value, making a difference in nursing students’ lives, being available and being involved in nursing students’ teaching and learning. Participants felt strongly about connecting, interacting and engaging with their nursing students through building good relationships. Participants stressed the importance of ‘engaging with students, the interaction between the students and the lecturer, and the interpersonal relationships that [they] build with students’ (Nurse Educators 5, 18, 20, 23). Nurse educators emphasised the importance of continuous monitoring of nursing students’ progress, and that providing positive feedback on a continuous basis contributes to students’ personal and professional development and growth. Being a role model, demonstrating care and respect, imparting knowledge and skills and sharing experience are ways to build nursing students’ confidence.

Nurse educators stated that they would implement this model in their classrooms after being asked: ‘Would you implement this model in your classroom?’ The majority of the participants responded with a ‘yes’. Participants’ non-verbal indicators revealed enthusiasm, willingness and an openness towards implementing this model. Participants indicated: ‘it would require of them to adapt to new teaching methods, receive adequate training, allocate sufficient time, and invest in proper planning. Responding to how it will influence their teaching practices’ (Nurse Educators 1, 5, 11, 27).

### Theme 2: benefits of implementing the model

Nurse educators responded to ‘how they think this model will benefit them and their nursing students if implemented’, whereas nursing students responded to *‘*what the advantages will be if this model is implemented in the classroom*’*. Participants emphasised that implementing this model in the classroom will be beneficial to different stakeholders in the nursing profession. Sub-themes include benefits for nurse educators, nursing students as well as other stakeholders.

Benefits for nurse educators revealed that reflection helps them to better understand nursing students, if they are coping and it enhances the quality of teaching. Nurse educators admitted that they would welcome feedback from their nursing students on their teaching practices. In other words, nursing students should participate in peer assessments on a continuous basis and not only twice a year as stipulated by most of the NEIs. Facilitating presence through guided reflection needs to be internalised and become a way of being. A participant responded: *‘*the rest of your life you need to practice presence and it can never stop’ (Nurse Educator 1). This will contribute to the professional growth and development for nurse educators by establishing a lifelong learning orientation. For learning to take place, it is emphasised that nurse educators need to create interesting classrooms for nursing students to engage in active participation.

Implementing this model will be beneficial to nursing students. Participants responded that nursing students will learn about their learning by becoming active participants in the learning process through continuous involvement and interaction. One participant stated: *‘*the most important thing about reflection, [is that] it enables the student to learn about their learning’ (Nurse Educator 1). Nursing students will enjoy learning because they will be motivated and able to take responsibility for their own learning, enabling them to become lifelong learners. Participants responded by noting that implementing this model will also increase participation and engagement; active and improved learning; improved self-confidence and the ability to manage problems in practice. Furthermore, equipping nursing students with reflective practices will not only ensure quality nurses but enable them to become critical thinkers. Their decision-making skills will be improved, resulting in them becoming independent, creative and innovative practitioners through integrating theory and practice, enabling them to implement evidence-based practices.

Benefits for all stakeholders, including nurse educators, nursing students and patients were highlighted. Nurse educators reported that they are empowered by participating in continuous professional development programmes to ensure that they stay abreast of any new developments; through this, they can empower nursing students. Participants emphasised that for learning to occur, it is important to build good and positive relationships with nursing students. Positive relationships consist of understanding, connecting, interacting and being open with each other. Nurse educators and nursing students should regard each other as unique human beings. When imprementing this model, practising presence through reflection will improve the mental well-being of nurse educators, nursing students and patients, improve the quality of care and enhance professional fulfilment. Participants emphasised that the implementation of this model in the undergraduate nursing programme at accredited NEIs will improve the quality of care patients will receive. One participant emphasised *‘*I think the model is excellent for both students and lecturers, [and]. . .will add to. . .quality patient care*’* (Nurse Educator 10). Implementing this model will bring presence to the forefront as nursing students will internalise and practice presence as part of their being. Nursing students will be more aware of what they are supposed to do and not merely focus on passing the qualification.

### Theme 3: limitations of the model

The findings revealed limited time, a lack of resources and resistance to change to be limitations that could impede the successful implementation of the model. In referring to limited time availability, participants responded ‘it will be time-consuming when implemented for the first time’ (Nurse Educators 3, 4, 8, 28). However, when nurse educators have internalised the practice of facilitating presence through guided reflection into their teaching and learning, it will become a way of doing. Presenting large amounts of content in a specified time frame can also be a hindrance to implementing this model. A lack of resources is strongly verbalised as the second limitation of implementing this model. Participants noted *‘*the lack of inadequate resources for teaching*’* (Nurse Educators 10, 13, 26, 34; Nursing Student 26). ‘Inadequate resources’ refers to insufficient equipment such as manikins for practical demonstrations; shortage of staff such as lecturers and clinical mentors; a lack of support from the NEIs such as management; and nursing students not being able to use devices such as cell phones or tablets for research purposes. These limitations were expressed in the responses of several participants: *‘*we must have all the relevant resources for teaching to take place’ (Nurse Educator 9), ‘the most important thing is the availability of resources for students*’* (Nurse Educator 13), and *‘*we might have limited resources in terms of lecturers’ (Nursing Student 26). Large classes could also impede the successful implementation of this model. With large classes, nurse educators would be unable to interact with all nursing students, which could prevent the early detection of learning problems. Participants responded: *‘*it will be difficult to be able to interact with everyone’ (Nurse Educator 7). Also, *‘*with large classes of nursing students, you wouldn’t be able to know [what] learning problems [are being experienced]’ (Nursing Student 26). The third limitation of implementing the model is resistance to change. When nurse educators are not open to new practices or refuse to adapt to better ways of teaching and education, it could hinder the successful implementation of the model. Participants emphasised: ‘there might be some resistance to change, resistance to adapt to better ways of teaching, and resistance to change for the adaptation of the new model’ (Nurse Educators 20, 23; Nursing Student 26).

### Theme 4: pre-existing conditions needed for the implementation of the model

Nurse educators were asked to ‘explain situations where they would apply this model’ and ‘What conditions need to exist to implement this model?’ Nursing education environment, staff development programmes, and stakeholder collaboration and support emerged as sub-themes.

The nursing education environment consists of the classroom where knowledge is obtained and clinical practice where skills are acquired. Nurse educators must create a conducive teaching–learning environment for nursing students. The classroom is where it all begins, and nurse educators need to implement this model from the first day that nursing students enter the nursing profession and it should be carried throughout their four years of training. Participants stated: ‘If the model is implemented throughout the four years, nursing students will become more competent and confident’ (Nurse Educator 28). It should also be implemented in clinical practice during the clinical accompaniment of nursing students. Nurse educators and preceptors should join hands and strive together to facilitate presence through reflective practices. This can be achieved through the proper orientation of nursing students in the clinical setting. Two participants mentioned that ‘it will also be beneficial to implement this model in the community’ (Nurse Educators 3, 8). It is important because nursing students are required to work community hours and participate in community projects. By utilising reflection, nursing students will be better equipped to work in communities and provide health education.

Staff development programmes are crucial for the improvement of teaching practices. Participants emphasised the importance of attending in-service training before implementation of this model to ensure a complete understanding of the model first before applying it in their classrooms. All stakeholders need to be involved. Participants also suggested that ‘it can form part of a teaching–learning project where nurse educators are invited to attend. . .in-service training for self-enrichment and improvement of teaching practices’ (Nurse Educators 1, 2, 16). One participant also emphasised the importance of ‘continuous follow-up with nurse educators after in-service training to encourage continuation in the implementation of the model’ (Nurse Educator 1).

Stakeholder support and collaboration occurs among nurse educators, nursing students and management of the NEI. Nurse educators as collaborators are responsible for building partnerships with relevant stakeholders. Participants revealed the importance of ‘the buy-in of all nurse educators, nursing students and management to ensure the successful implementation of this model (Nurse Educators 1, 2. 3, 8, 13, 15). Nurse educators are regarded as role models in the sense that they need to practice what they preach by setting an example of how to practice presence. One participant stated how important it is that ‘nurse educators. . .demonstrate positive attitudes towards nursing students*’* (Nurse Educator 17).

### Theme 5: recommendations for further development of the model

During the final interview question, participants were provided with an opportunity to share their ideas, opinions and viewpoints on ‘what can be included to make this model more useful, relevant and effective’. The overall feeling was that participants were satisfied with the layout, structural components and visualisation of the model. However, some of the participants voiced their concerns about the gaps identified which gave rise to theory–practice integration and inclusion of other stakeholders as sub-themes. For theory–practice integration, participants suggested the model be implemented in the clinical practice setting as well. However, this model focuses on nursing education specific to the classroom. Stakeholders to be included comprise patients, parents of nursing students and the community. Participants recommended that it is necessary to acknowledge the reflections of patients; to involve the parents as well as the community of nurses.

## Discussion and recommendations

Both nurse educators and nursing students demonstrated a clear understanding of the classroom implementation of the model although participants suggested that training should be provided to ensure a comprehensive understanding of the model before it is implemented. Attributes of the nurse educator as evident in the findings correspond with the presence attributes embedded in the model. These attributes highlight being a role model by sharing experiences [4, 22], connecting and encouraging continued participation [5, 23, 24], and establishing meaningful, open and honest interactions with nursing students for relationship-building [25, 26]. Additionally, introducing strategies for reflective practices in the classroom will assist nurse educators to transform their teaching practices [27] which will facilitate presence. Reflective practices require active skills, interpersonal approaches, techniques and adequate resources used by the nurse educator [28]. These were addressed in the process phase of the model, where the author provides nurse educators with strategies to implement new teaching methods, adequate time management and proper planning, as is evident in the findings. Therefore, the feasibility of implementing this model in the classroom was reached.

It was evident that the implementation of this model at accredited NEIs will have numerous benefits for nurse educators, nursing students and patients. Presence through reflection requires establishing meaningful relationships with nursing students where for learning itself to become meaningful, the nurse educator can challenge, enable and support nursing students. Furthermore, presence and reflective practices contribute to continuous professional development and lifelong learning [29–31]; personal and professional satisfaction [23, 32]; physical and mental well-being for the nurse educator, nursing student and patient [23, 32]; improved patient outcomes and, ultimately, quality nursing care.

Presence can be hampered by environmental factors such as time constraints and lack of resources [26]. Time constraints impact the nurse educator’s ability to be present with their nursing students owing to scheduled class times and, therefore, they need to allocate time to specific tasks and activities during the lesson presentation to reach learning outcomes. In addition, NEIs need to ensure adequate resources such as sufficient equipment and staff for teaching nursing students, including the selection of appropriate learning activities and interactive teaching strategies in large class settings to facilitate presence [4, 31]. Mulryan-Kyne (2010) emphasises that utilising more active teaching approaches, careful planning, commitment and the provision of adequate and appropriate resources is advantageous for teaching large class groups of nursing students. Utilising teaching strategies such as problem-based learning and small group discussions where nursing students can ask frequent questions optimises the opportunities for nursing student engagement, especially in large class settings [33]. In addition, NEIs need to support and promote a reflective practice environment and culture which will contribute to quality teaching practices [34] where presence can evolve.

Pre-existing conditions needed for the successful implementation of this model revealed a conducive teaching–learning environment where nurse educators are invested and committed to bringing about change in their nursing students. Presence requires a conducive teaching–learning environment that is safe and supported [26, 35], where the nurse educator ensures collaboration and shared meaning-making, engages in ongoing communication and reflection, and encourages continued participation [25]. Nurse educators require support from faculties in the form of attending professional development programmes consisting of content development, learning activities, teaching strategies and assessment techniques [31] fundamental for enhancing nurse educators’ knowledge and skills to facilitate presence. These development programmes can be in the form of in-service training sessions, workshops or seminars to explain the model and can assist nurse educators with the successful implementation thereof.

## Limitations

The study was conducted at a rural university and nursing college in the North West Province. Virtual World Café sessions were planned to be conducted at four research sites, but only three research sites were included due to strikes taken place at the fourth site. Only 4th-year nursing students were included and by the time informed consent had been obtained and data collection commenced, strikes were taking place; further, many students were completing their final practical hours and had insufficient time to participate. Thus only 34 nursing students participated in the three virtual World Café sessions. Data was provided to all participants, some reported that they could not attend owing to technical issues such as unstable internet connection and inability to connect.

Owing to the COVID pandemic and the associated lockdown restrictions, health and safety protocols were implemented to protect the safety of participants by preventing any face-to-face interactions. This forced the adaptation of data collection methods from face-to-face to online methods. The online environment brought along its own challenges:

- Using Zoom with disabled webcams: Participants who disabled their webcams limited the author’s reflection on non-verbal indicators of body language, eye contact, tone of voice, facial expression and general appearance when they participated or when a co-participant shared their views.
- Using Zoom with open microphones: Keeping all microphones on during the interviews sometimes made it difficult to hear participants’ responses owing to background noise. Participants had to be repeatedly reminded to mute themselves while they were not speaking, which led to unnecessary interruptions and frustrations.
- Technological constraints such as connectivity problems and unstable internet connection also contributed to problems with clearly hearing participants. Disruptive factors included lost signal, the need to repeat questions and answers, and background interference while participants were sharing.

## Conclusions

The results of the empirical phase of the research enabled the researchers’ to evaluate and refine the developed model, achieving the aim of this study. Valuable feedback was received from participants. Therefore, the findings from this study contribute to the substantial deepening of the body of knowledge on nursing education and training, practice and research in the South African nursing education domain as well as in the international nursing education context. Incorporating the developed model into the curriculums of undergraduate, postgraduate and continuous professional development programmes will not only build the body of knowledge but will also increase nursing students’ awareness of presence.

Faculty to develop in-service training programmes in which this model can be presented to nurse educators before implementation thereof. In addition, further research is needed for the operationalisation and validation of the model for nurse educators to facilitate presence in large class settings through reflective practices; the development of a model to facilitate presence through reflective practices in the clinical practice environment that will contribute to theory–practice integration; as well as development of guidelines to operationalise the model within other disciplines.

## Data Availability

The datasets used for the current study are available from the corresponding author upon request.

## Abbreviations

HREC: Health Research Ethics Committee
NEI(s): Nursing Education Institution(s)
RDGC: Research Data Gatekeeper Committee
SANC: South African Nursing Council

## References

[1] South African Nursing Council. 2005. The philosophy and policy of the South African Nursing Council with regard to professional nursing education. http://www.sanc.co.za/pdf/Learner%20docs/Philosophy%20and%20Policy%20of%20SANC%20re%20Professional%20Nursing%20Education.pdf Date of access: 06 Dec. 2019.

[2] South African Nursing Council. 2014. Competencies for a nurse educator. Pretoria. http://www.sanc.co.za Date of access: 11 Dec. 2019.

[3] Boeck PR (2014). Presence: a concept analysis. SAGE J.

[4] Kostovich CT, Clementi PS (2014). Nursing presence. Putting the art of nursing back into hospital orientation. J Nurses Prof Dev.

[5] Baart A, Timmerman G, Du Plessis E (2021). Research methods for research on presence. Reflecting on presence in nursing: a guide for practice and research.

[6] Du Plessis E, Beurskens E. 2021. Being present: an account of an exposure. In: Du Plessis, E., ed. (2021) Reflecting on presence in nursing: a guide for practice and research. Newcastle upon Tyne: Cambridge Scholars Publishing. pp. 20–39.

[7] Oukouomi Noutchie C. 2019. The cultivation of caring presence in nurses: a systematic review. South Africa: NWU (Mini-dissertation: Masters).

[8] Hatlevik IKR (2011). The theory-practice relationship: reflective skills and theoretical knowledge as key factors in bridging the gap between theory and practice in initial nursing education. J Adv Nurs.

[9] Mulryan-Kyne C (2010). Teaching large classes at college and university level: challenges and opportunities. Teach High Educ.

[10] Taylor-Haslip V. 2013. The lived experience of caring presence for nursing faculty and nursing students. New York: The City University of New York. (Dissertation: PhD). https://scholar.google.com/scholar?hl=en&as_sdt=0%2C5&q=Taylor-Haslip%2 C+V.++2013.++The+lived+experience+of+caring+presence+for+nursing+faculty+and+nursing+students.+&btnG Date of access: 10 Dec. 2019.

[11] Froneman K, du Plessis E, van Graan AC (2022). Conceptual model for nurse educators to facilitate their presence in large class groups of nursing students through reflective practices: a theory synthesis. BMC Nurs.

[12] Gray JR, Grove SK, Sutherland S (2017). Burns and Grove’s. The practice of nursing research. Appraisal, synthesis and generation of evidence.

[13] Chinn PL, Kramer MK (2018). Integrated theory and knowledge development in nursing.

[14] Botma Y, Greeff M, Muluadzi FM, Wright SCD (2022). Research in health sciences.

[15] Krueger RA, Casey MA (2015). Focus groups: a practical guide for applied research.

[16] Tates K, Zwaanswijk M, Otten R, van Dulmen S, Hoogerbrugge PM, Kamps WA, Bensing JM. Online focus groups as a tool to collect data in hard-to-include populations: examples from paediatric oncology. BMC Med Res Methodol. 2009;9(15). 10.1186/1471-2288-9-15. 10.1186/1471-2288-9-15 PMC2653071 19257883

[17] Grove SK, Burns N, Gray JR (2013). The practice of nursing research. Appraisal, synthesis and generation of evidence.

[18] Polit DF, Beck CT (2020). Nursing research: generating and assessing evidence for nursing practice.

[19] Brown J, Isaacs D (2005). The world café: shaping our futures through conversations that matter.

[20] Koen MP, Du Plessis E, Koen V. 2014. Data analysis: the world café. (In De Chesnay, M. ed. Nursing research using data analysis. Qualitative designs and methods in nursing. Chapter 12). http://www.springerpub.com/nursing-research-using-data-analysis.html Date of access: 3 September 2018.

[21] Creswell JW, Creswell JD (2018). Research design: qualitative, quantitative, and mixed methods approaches.

[22] Bozdoğan Yeşilot S, Öz F (2016). Nursing presence: a theoretical overview. J Psychiatric Nurs.

[23] Bozdoğan Yeşilot, s., Öz F. 2016. Nursing presence: a theoretical overview. *Journal of Psychiatric Nursing*, 7(2):94–99. doi:10.5505/phd.2016.96967.

[24] Zhan Z, Mei H (2013). Academic self-concept and social presence in face-to-face and online learning: perceptions and effects on students’ learning achievement and satisfaction across environments. Comput Educ.

[25] Caudle LA (2013). Using a sociocultural perspective to establish teaching and social presences within a hybrid community of mentor teachers. Adult Learn.

[26] McMahon MA, Christopher KA (2011). Toward a mid-range theory of nursing presence. Nurs Forum.

[27] Belvis E, Pineda P, Armengol C, Moreno V (2013). Evaluation of reflective practice in teacher education. Eur J Teacher Educ.

[28] Moreno JM, Sanyal KA, Firoozmand F, Rutter P, Harder MK (2020). Reflective practices in community development: a grounded analysis. Systemic Pract Action Res.

[29] Bulman C, Lathlean J, Gobbi M (2014). The process of teaching and learning about reflection: research insights from professional nurse education. Stud High Educ.

[30] Edwards S (2017). Reflecting differently. New dimensions: reflection-before-action and reflection-beyond-action. Int Pract Dev J.

[31] Gurley LE (2018). Educators’ preparation to teach, perceived teaching presence, and perceived teaching presence behaviours in blended and online learning environments. Online Learn.

[32] Stockmann C (2018). Presence in the nurse–client relationship: an integrative review. Int J Hum Caring.

[33] Exeter DJ, Ameratunga S, Ratima M, Morton S, Dickson M, Hsu D, Jackson R (2010). Student engagement in very large classes: the teachers’ perspective. Stud High Educ.

[34] Naicker K, van Rensburg GH (2018). Facilitation of reflective learning in nursing: reflective teaching practices of educators. Afr J Nurs Midwifery.

[35] Turpin RL (2014). State of the science of nursing presence revisited: knowledge for preserving nursing presence capability. Int J Hum Caring.

